# Hope level of primary caregivers of children with epilepsy: current status and influencing factors

**DOI:** 10.3389/fped.2025.1741576

**Published:** 2026-01-29

**Authors:** Rui Wu, Jinfang Zhou

**Affiliations:** Department of Neurology, Children’s Hospital of Nanjing Medical University, Nanjing, Jiangsu, China

**Keywords:** care, caregivers, children, epilepsy, hope, nursing

## Abstract

**Background:**

Primary caregivers of children with epilepsy bear long-term care responsibilities and face multiple pressures, which affect their hope level. This survey aimed to evaluate the hope level and associated factors of primary caregivers of children with epilepsy, to provide insights for clinical nursing care.

**Method:**

A cross-sectional survey was conducted among primary caregivers of children with epilepsy in a tertiary grade A children's hospital in China. Data were collected using a general information questionnaire and the hope scale, and analyzed via univariate analysis, Pearson correlation analysis, and multiple linear regression analysis.

**Results:**

A total of 248 primary caregivers were included. The overall hope level of caregivers was moderate (total score: 26.25 ± 4.92), with the Intimate Relationship dimension having the lowest score (score rate: 50.44%). Pearson correlation analysis showed that age (*r* = 0.382), educational level (*r* = 0.459), and monthly household income (*r* = 0.417) were positively correlated with hope level (all *P* < 0.001), while the three scale dimensions (Positive Attitude, Positive Action, Intimate Relationship) all had strong positive correlations with total hope score (all r > 0.85, *P* < 0.001). Multiple linear regression showed that age (*β* = 0.193, *P* < 0.001), marital status (*β* = −2.876, *P* < 0.001), residence (*β* = −3.152, *P* < 0.001), educational level (*β* = 1.268, *P* < 0.001), and monthly household income (*β* = 1.054, *P* < 0.001) were independent influencing factors of hope level (model fit: *R*^2^ = 0.482, adjusted *R*^2^ = 0.467).

**Conclusion:**

The hope level of primary caregivers of children with epilepsy was moderate. Younger age, divorced/widowed status, rural residence, lower educational level, and poorer economic conditions were the negative influencing factors. Targeted interventions should be implemented to elevate caregivers’ hope levels.

## Introduction

Epilepsy, a globally prevalent chronic neurological disorder characterized by prolonged course and recurrent seizures (1), not only impairs the physical and mental health and development of affected children but also imposes long-term care pressure on families (2). The prevalence of epilepsy among children in China is approximately 3%–6% (3, 4). Children with epilepsy require long-term medication to control their condition, along with continuous daily care and health monitoring. This makes primary caregivers an indispensable core part of the disease management process for these children (5). These caregivers face multiple stressors, including uncertain prognosis, persistent treatment costs, and social stigma (6, 7), and their mental status directly influences care quality, treatment adherence, and ultimately the rehabilitation outcomes of the children.

Hope, defined as an individual's positive future expectations and willingness to act amid difficulties (8–11), is a critical psychological resource for coping with stress and maintaining mental health. For caregivers of children with epilepsy, a favorable hope level supports positive caregiving mindsets and effective stress management, while low hope may lead to burnout, reduced coping capacity, and compromised family function (12–14). Thus, understanding the hope level of these caregivers and its influencing factors is crucial for safeguarding their mental health and optimizing care quality.

Existing research on caregivers of children with chronic diseases has primarily focused on negative emotions such as anxiety and depression (15–19), with a notable gap in specialized, systematic investigations into the hope level (a positive psychological resource) of primary caregivers of children with epilepsy. This study therefore aims to examine the current status of hope among these caregivers and identify its key influencing factors, providing a scientific basis for improving caregiver mental health and constructing comprehensive family support systems.

## Methods

### Study design and ethical considerations

This study adopted a cross-sectional survey design and was conducted in a tertiary grade A children's hospital in China from October 2024 to September 2025. This study was approved by the Medical Ethics Committee of the participating tertiary grade A children's hospital (Ethics Approval No.: 202512029-1) and was conducted in strict accordance with the Declaration of Helsinki and the fundamental principles of medical research involving human subjects. Before the implementation of the survey, researchers who received unified training explained the study purpose, content, data usage, and privacy protection measures to all participants in detail to ensure they fully understood the research process. The written informed consent was obtained from all the included caregivers. For caregivers with low educational levels who were unable to complete the questionnaire independently, researchers read the questionnaire items in a “one-on-one” non-inductive manner and filled in the questionnaire based on the caregivers’ true responses.

### Sample size calculation

The sample size was determined using the sample size estimation formula for cross-sectional surveys (20): $n=Z_{\alpha /2}^{2}\times \;p(1-p)/\delta^{2}$, where *α* was set to 0.05 (two-tailed test) with the corresponding $Z_{\alpha /2}^{2}=1.96$; referring to relevant previous studies, the abnormal rate (p) of hope level among caregivers of children was approximately 35%, and the allowable error (δ) was set to 0.06. The initial estimated sample size was 168 cases. Considering the potential invalid questionnaires during the filling process (e.g., missing rate >20%, logical contradictions), the sample size was expanded by an invalid sample rate of 20%. Finally, the minimum required sample size was determined to be 202 cases to ensure the statistical power of the study results.

### Study participants

The study participants were primary caregivers of children with epilepsy who visited the participating hospital during the above survey period. The inclusion criteria were as follows: (1) Adults (aged ≥18 years) who lived with the child with epilepsy and undertook ≥80% of the child's daily care (e.g., diet, medication administration, rehabilitation training, medical accompaniment); (2) Children diagnosed with epilepsy through clinical symptoms, electroencephalography, and imaging examinations with a disease duration of ≥3 months; (3) Caregivers with basic language comprehension and communication abilities, capable of cooperating in completing questionnaire filling or interviews; (4) Caregivers who voluntarily participated in this study and signed the informed consent form. The exclusion criteria were: (1) Caregivers with severe mental illnesses (e.g., schizophrenia, major depressive disorder) or cognitive impairments, unable to accurately express their feelings; (2) Children with other severe organic diseases (e.g., malignant tumors, severe cerebral palsy) or hereditary metabolic diseases; (3) Caregivers in the acute phase of illness or within 1 month of experiencing a major life stress event (e.g., death of a relative, marital change) during the survey period. A convenient sampling method was adopted to recruit participants from the pediatric neurology outpatient department and inpatient ward of the participating hospital. Eligible caregivers were approached by trained researchers during their regular visits or hospitalization periods for their children's epilepsy treatment. The recruitment process continued until the required sample size (*n* = 202) was reached, with a final enrollment of 248 caregivers to account for potential invalid questionnaires.

### Research instruments

The following general information of child caregivers was collected: gender, age, marital status, place of residence, educational level, employment status, monthly household income, relationship with the child, and number of children.

Hope Level Scale: This scale was validated and published by Zhao et al (21), and has been specifically validated for use in both patients with chronic diseases and their caregivers, demonstrating suitability for evaluating the hope level of this dual population (22). It has good reliability and validity (Cronbach's *α* coefficient: 0.85–0.92; content validity index: 0.93). The scale consists of 12 items, divided into 3 dimensions: “Positive Attitude” (4 items: 1, 4, 7, 12), “Positive Action” (4 items: 2, 5, 10, 11), and “Intimate Relationship” (4 items: 3, 6, 8, 9). A 4-point Likert scale was used, with scores ranging from 1 to 4 (1 = “Strongly Disagree” to 4 = “Strongly Agree”). Among them, items 3 and 6 were reverse-scored items (i.e., 4 points for “Strongly Disagree” and 1 point for “Strongly Agree”). The total score of the scale ranges from 12 to 48 points; a higher score indicates a higher level of hope of the caregiver. According to the total score, the hope level was divided into 3 grades: 12–23 points for low level, 24–35 points for medium level, and 36–48 points for high level, providing a basis for subsequent status analysis and hierarchical intervention (23). In the present study, the internal consistency reliability of the scale was verified using our dataset, yielding a Cronbach's α coefficient of 0.87, which indicates good internal consistency and confirms the scale's applicability to our target population.

### Survey details

Before the implementation of the study, all investigators received unified training, covering the interpretation of the study protocol, explanation of questionnaire items, communication skills, data recording standards, and ethical considerations. Only those who passed the assessment (with a 100% qualification rate) could participate in the survey. The survey was conducted in a quiet consultation room or interview room in the hospital to avoid external interference. Researchers first checked the children's medical records to confirm that they met the inclusion criteria for study participants, then communicated with the caregivers to obtain informed consent. The time for filling out the questionnaire was controlled within 15–20 min; during the filling process, researchers needed to be present throughout to answer the caregivers’ questions about the questionnaire items in a timely manner (e.g., explanation of the definition of “short-term, medium-term, or long-term goals” in the “Positive Action” dimension), but no inductive prompts were allowed. After the questionnaires were collected, two researchers independently conducted data verification: (1) Checking the completeness of the questionnaires: questionnaires with ≤3 missing items and no missing key information were supplemented and completed through telephone follow-up; questionnaires with >3 missing items or obvious logical errors (e.g., “20 years old” but “widowed” was checked for marital status) were determined to be invalid and excluded; (2) A database was established using EpiData 3.1 software (EpiData Association, Odense, Denmark), and “double entry + consistency check” was implemented. Data with inconsistent entries were checked against the original questionnaires and corrected in a timely manner to ensure the accuracy of data entry and lay a foundation for subsequent statistical analysis. For questionnaires with ≤3 missing items, the missing values were supplemented through telephone follow-up with caregivers. Questionnaires with >3 missing items or logical contradictions were excluded from the final analysis. No statistical imputation methods were applied, as the proportion of missing data was low (3.2%, 8/248 questionnaires with missing items) and fully supplemented via follow-up. All 248 caregivers were included in the final statistical analysis after data supplementation.

### Statistical analysis

SPSS 26.0 statistical software (IBM Corp., Armonk, NY, USA) was used for data processing. All statistical tests were two-tailed tests, with a test level of *α* = 0.05. First, descriptive analysis of data was conducted: measurement data conforming to a normal distribution were expressed as “mean ± standard deviation”, such as the total score of caregivers’ hope level and scores of each dimension; categorical data were expressed as “number of cases (constituent ratio, *n*/%)”, such as the gender distribution and marital status composition of caregivers. Second, univariate analysis was performed: independent samples t-test was used for two groups of measurement data (e.g., hope level scores of caregivers of different genders); one-way analysis of variance (ANOVA) was used for multiple groups of measurement data (e.g., hope level scores of caregivers in different age groups or with different educational levels). If there were statistically significant differences between groups, LSD-t test was further used for pairwise comparison. Subsequently, correlation analysis was carried out: Pearson correlation analysis was used to explore the correlation strength and direction between continuous variables or ordinal categorical variables (such as caregivers’ age, educational level, and monthly household income) and the total score of hope level. Finally, multivariate analysis was conducted: taking the total score of hope level as the dependent variable, variables with *P* < 0.10 in the univariate analysis (e.g., age, marital status, place of residence) were included in the multiple linear regression analysis. Stepwise regression method (entry criterion *α* = 0.05, elimination criterion *α* = 0.10) was used to screen the independent factors affecting caregivers’ hope level. The regression coefficient, standardized regression coefficient, and 95% confidence interval were used to reflect the influence degree of each factor. Meanwhile, R² and adjusted R² were used to evaluate the goodness of fit of the regression model. There was statistical difference when *P* < 0.05.

## Results

### Baseline characteristics and univariate analysis of caregivers’ hope level

A total of 248 primary caregivers of children with epilepsy were included in this study, and the results of univariate analysis on their baseline characteristics and hope level are presented in Table 1. Gender had no significant effect on hope level (*P* = 0.217); the score of female caregivers was slightly lower than that of male caregivers, but the difference was not statistically significant. Among the age groups, caregivers in the 20–30 years old group had the lowest hope level, while those in the 41–50 years old group had the highest, with a statistically significant difference between groups (*P* < 0.001), suggesting that younger caregivers are more likely to have lower hope levels. Marital status, place of residence, educational level, and monthly household income all had significant impacts on hope level (all *P* < 0.001): married caregivers had significantly higher scores than divorced/widowed ones, and urban caregivers had higher scores than rural ones; educational level and monthly household income showed a gradient association, i.e., the higher the educational level and the better the income level, the higher the caregivers’ hope level. In addition, employment status, number of children, and relationship with the child had no statistically significant effects on hope level (all *P* > 0.05).

**Table 1:** Univariate analysis of baseline characteristics and hope level among 248 primary caregivers of children with epilepsy.

Baseline characteristic	Grouping	Sample size	Hope level score	Test statistic	*P*
Gender	Male	72	26.89 ± 4.71	t = 1.238	0.217
	Female	176	25.93 ± 5.02		
Age (years)	20–30	58	23.15 ± 4.28	F = 10.562	<0.001
	31–40	112	27.02 ± 4.53		
	41–50	65	28.37 ± 4.19		
	≥51	13	27.81 ± 3.96		
Marital status	Married	195	27.93 ± 4.35	F = 18.245	<0.001
	Divorced/Widowed	53	22.06 ± 4.87		
Residence	Urban	163	28.51 ± 4.06	t = 7.623	<0.001
	Rural	85	23.07 ± 5.14		
Educational level	Junior high school and below	89	22.83 ± 4.79	F = 22.317	<0.001
	Senior high school/vocational secondary school	95	26.58 ± 4.25		
	College and above	64	29.91 ± 3.86		
Employment status	Employed	102	27.16 ± 4.59	t = 2.015	0.054
	Unemployed	146	25.98 ± 5.11		
Monthly household income (RMB)	≤5,000	108	22.59 ± 4.98	F = 19.684	<0.001
	5,001–10,000	86	26.93 ± 4.12		
	>10,000	54	29.07 ± 3.75		
Number of children	1	123	25.87 ± 5.03	F = 1.326	0.267
	2	96	26.71 ± 4.78		
	≥3	29	27.05 ± 4.51		
Relationship with the child	Parents	201	26.58 ± 4.89	F = 0.874	0.455
	Grandparents	42	25.31 ± 5.26		
	Other relatives	5	24.92 ± 5.43		

Statistical methods: Independent samples t-test was used for gender and residence; one-way analysis of variance (ANOVA) was used for other categorical variables. A *P* value < 0.05 was considered statistically significant.

### Current status of caregivers’ hope level

The overall hope level of the 248 caregivers was at a moderate level, with a total score of 26.25 ± 4.92 and a score rate of 54.69% (Table 2). From the perspective of each dimension, the score rates from high to low were as follows: Positive Attitude (57.81%), Positive Action (55.81%), and Intimate Relationship (50.44%). Among them, the Intimate Relationship dimension had the lowest score rate, which was 4.25 percentage points lower than the overall level. Further analysis showed that items reflecting positive cognition towards difficulties in the Positive Attitude dimension had relatively high scores, while reverse-scored items reflecting loneliness and worry about the future in the Intimate Relationship dimension had relatively low scores.

**Table 2:** Dimension scores of hope among 248 primary caregivers of children with epilepsy.

Scale dimension	Number of items	Maximum score of dimension	Actual score (x ± s)	Score rate (%)
Positive Attitude	4	16	9.25 ± 1.87	57.81
Positive Action	4	16	8.93 ± 1.92	55.81
Intimate Relationship	4	16	8.07 ± 2.15	50.44
Total Scale Score	12	48	26.25 ± 4.92	54.69

Score rate = (Mean actual score/Maximum score of dimension) × 100%. A higher score rate indicates a higher level of hope in the corresponding dimension.

### Pearson correlation analysis results

The results of Pearson correlation analysis are shown in Table 3. Age, educational level, and monthly household income all had a significant positive correlation with hope level (all *P* < 0.001). Among them, educational level had the strongest correlation (r = 0.459), followed by monthly household income (r = 0.417) and age (*r* = 0.382). This indicates that the older the age, the higher the educational level, and the better the economic conditions, the higher the caregivers’ hope level. From the perspective of internal dimensions of the scale, Positive Attitude, Positive Action, and Intimate Relationship all had a very strong positive correlation with the total score of hope level (all *r* > 0.85, all *P* < 0.001), and the Positive Attitude dimension made the greatest contribution. This suggests that the three dimensions together constitute the core elements of hope level, among which positive cognition plays the most prominent role.

**Table 3:** Pearson correlation analysis between baseline characteristics and hope level among 248 primary caregivers of children with epilepsy.

Variable	Correlation coefficient (r)	*P*
Age	0.382	<0.001
Educational level	0.459	<0.001
Monthly household income	0.417	<0.001
Score of positive attitude dimension	0.893	<0.001
Score of positive action dimension	0.876	<0.001
Score of intimate relationship dimension	0.852	<0.001

### Multiple linear regression analysis results

To screen for independent factors affecting hope level, variables with *P* < 0.10 in the univariate analysis (age, marital status, place of residence, educational level, employment status, and monthly household income) were included in the multiple linear regression analysis, and the variable assignment is shown in Table 4.

**Table 4:** Variable assignment for multiple linear regression analysis of hope level among primary caregivers of children with epilepsy.

Independent variable	Assignment method
Age	Entered as actual age (years)
Marital status	0 = Married, 1 = Divorced/Widowed
Residence	0 = Urban, 1 = Rural
Educational level	1 = Junior high school and below, 2 = Senior high school/vocational secondary school, 3 = College and above
Employment status	0 = Unemployed, 1 = Employed
Monthly household income	1 = ≤5,000 RMB, 2 = 5,001–10,000 RMB, 3 = >10,000 RMB
Dependent variable	Total hope score (12–48 scores)

The multiple linear regression model had a good goodness of fit (*R*^2^ = 0.482, adjusted *R*^2^ = 0.467, *P* < 0.001) and could explain 46.7% of the variation in hope level (Table 5). The results showed that age, educational level, and monthly household income were positive predictors of hope level (all *P* < 0.001), while marital status (divorced/widowed) and place of residence (rural) were negative predictors (all *P* < 0.001).

**Table 5:** Results of multiple linear regression analysis of hope level among 248 primary caregivers of children with epilepsy.

Independent variable	Regression coefficient (β)	Standard error (SE)	Standardized regression coefficient (β’)	t	*P*	95% confidence interval
Constant term	15.287	1.893	–	8.075	<0.001	[11.562, 19.012]
Age	0.193	0.045	0.215	4.289	<0.001	[0.105, 0.281]
Marital status (divorced/widowed = 1)	−2.876	0.632	−0.238	−4.551	<0.001	[−4.118, −1.634]
Residence (rural = 1)	−3.152	0.597	−0.267	−5.280	<0.001	[−4.328, −1.976]
Educational level	1.268	0.271	0.293	4.679	<0.001	[0.736, 1.799]
Monthly household income	1.054	0.283	0.229	3.724	<0.001	[0.498, 1.610]

Model fit: *R*^2^ = 0.482, adjusted *R*^2^ = 0.467, F = 32.158, *P* < 0.001.

## Discussion

This study found that the overall hope level of primary caregivers of children with epilepsy was moderate, a finding consistent with previous research on caregivers of children with diseases (24–26). Specifically, the persistent burden of long-term caregiving is known to constrain the positive psychological resources available to this population. In terms of dimensional scores, the *Intimate Relationship* domain yielded the lowest score rate, which was markedly below the overall average. Notably, reverse-coded items assessing feelings of loneliness and apprehensions about the future also exhibited relatively low scores. This pattern suggests that caregivers experience a pronounced deficit in emotional support throughout the caregiving journey.

A plausible explanation for this observation lies in the unique challenges posed by pediatric epilepsy: the condition's unpredictable onset and uncertain prognosis necessitate that caregivers maintain sustained high levels of vigilance. This chronic state of alertness diminishes the time and energy caregivers can allocate to nurturing interpersonal relationships (27, 28). Furthermore, societal stigma associated with epilepsy may deter caregivers from engaging in social interactions, thereby exacerbating their sense of isolation and impeding their capacity to develop positive future-oriented expectations (29).

Univariate analysis and multiple linear regression analysis consistently confirmed that age, marital status, and residence are key factors affecting caregivers’ hope level. Younger caregivers had the lowest hope level, while age served as a positive predictor of hope level. This may be because older caregivers possess more life experience and coping strategies, enabling them to better adjust their mindset when facing care difficulties (30); in contrast, younger caregivers are often in the early stages of life and career development, lacking experience in addressing long-term care challenges, which makes them more prone to helplessness. Divorced/widowed caregivers showed significantly lower hope level than married caregivers, with marital status (divorced/widowed) acting as a negative predictor. With regard to gender differences, our univariate analysis revealed no statistically significant variation in hope level between male and female caregivers (male: 26.89 ± 4.71 vs. female: 25.93 ± 5.02, *P* = 0.217). This may be attributed to the fact that both genders bear comparable long-term care responsibilities and psychological pressure when caring for children with epilepsy, which offsets potential gender-based differences in positive psychological resources such as hope. Married caregivers can obtain emotional comfort and practical assistance from their spouses to share care pressure, whereas single caregivers (divorced/widowed) must bear all care responsibilities alone, leading to easier accumulation of psychological pressure (31). In terms of residence, rural caregivers had lower hope level than urban caregivers, and rural residence was a negative predictor. The primary reason may be that rural areas have relatively insufficient medical and social support resources, caregivers have limited access to professional guidance on epilepsy treatment and rehabilitation, and struggle to obtain timely help when encountering care problems, thus reducing their confidence in the future (32).

This study revealed that educational level and monthly household income were strongly positively correlated with caregivers’ hope level, and both emerged as positive predictors in multiple linear regression analysis. This suggests that higher educational level and better economic conditions correspond to higher caregiver hope level. For educational level, caregivers with higher education have stronger abilities to acquire and understand information, they can more effectively learn epilepsy-related treatment and care knowledge through medical manuals, professional websites, and other channels, and form a more rational understanding of disease prognosis, thereby reducing unnecessary anxiety. In terms of economic conditions, epilepsy requires long-term medication and regular follow-up examinations, imposing a heavy economic burden on families (33). Caregivers with better economic conditions can better cope with disease-related economic pressure, avoid the dilemma of treatment interruption due to insufficient funds, and thus maintain greater confidence in the child's recovery process (34).

Pearson correlation analysis showed that the three dimensions of the hope scale (Positive Attitude, Positive Action, Intimate Relationship) all had a very strong positive correlation with the total hope score, and the Positive Attitude dimension contributed the most. This confirms that the three dimensions are closely interconnected and jointly constitute the core of caregivers’ hope level, with positive cognition of difficulties playing a leading role. A positive attitude can drive caregivers to take active actions (such as actively cooperating with treatment and formulating care plans), and these positive actions further enhance their sense of control over the disease, thereby boosting their confidence in the future (26). At the same time, a positive attitude helps caregivers maintain good interpersonal relationships and obtain more emotional support, which in turn strengthens their positive cognition (35). This interactive relationship implies that when improving caregivers’ hope level, efforts should focus on cultivating their positive attitude toward the disease, while also paying attention to the coordinated improvement of all three dimensions.

The findings of this study are generally consistent with previous research on caregivers of children with chronic diseases, while also reflecting characteristics specific to the epilepsy population. For example, previous studies (36, 37) have identified marital status and economic conditions as important factors affecting caregivers’ psychological status, and this study further confirms that these factors also significantly impact the hope level of caregivers of children with epilepsy, quantifying their influence through regression coefficients. Additionally, this study highlights that the Intimate Relationship dimension is the weakest link in caregivers’ hope level, filling the research gap regarding the specific dimensional performance of hope level in this population. The practical significance of this study lies in clarifying the key factors affecting the hope level of caregivers of children with epilepsy, which provides a targeted basis for formulating nursing intervention measures, and helps improve caregivers’ psychological status, optimize care quality, and ultimately promote the recovery of children with epilepsy.

Based on the research results, the following targeted nursing countermeasures are proposed to improve the hope level of primary caregivers of children with epilepsy: firstly, strengthen psychological support for vulnerable groups—for younger caregivers, conduct regular psychological counseling and experience exchange sessions, invite experienced older caregivers to share coping strategies, and help younger caregivers enhance their ability to cope with care pressure; for divorced/widowed caregivers, establish peer support groups to encourage mutual communication and assistance, and provide one-on-one psychological guidance when necessary to alleviate their sense of loneliness; and for rural caregivers, use mobile health (mHealth) platforms to regularly push epilepsy-related treatment, care, and rehabilitation knowledge, as well as set up online consultation channels with medical professionals to promptly address their care-related questions (38). Secondly, improve the accessibility of medical and social support resources by collaborating with local governments and social organizations to provide economic assistance for low-income caregiver families (such as reducing medical expenses or offering rehabilitation training subsidies) in order to alleviate their economic pressure, while establishing a “hospital-community-family” linked support system, arranging for community nurses to conduct regular home visits, providing on-site guidance on child care, and helping caregivers connect with local social support resources (such as community care services and volunteer teams) (39). Thirdly, carry out targeted health education and positive attitude training: develop personalized health education materials based on caregivers’ educational level (e.g., picture books for those with lower education, professional manuals for those with higher education), explain epilepsy prognosis and treatment knowledge in an easy-to-understand manner to help caregivers establish a rational understanding of the disease, and offer positive psychology training courses to teach caregivers skills such as positive cognitive reconstruction and emotional regulation, guiding them to face care difficulties proactively and form a positive attitude toward the future (40). Fourthly, focus on improving the Intimate Relationship dimension by organizing family interaction activities to encourage more communication between caregivers and family members, promoting family members to participate in the care process together to enhance caregivers’ sense of family support, and conducting social adaptation training for caregivers to help them correctly understand social prejudice against epilepsy and encourage them to maintain normal interpersonal relationships, thereby enabling them to obtain more external emotional support (41).

## Strengths and limitations

Several strengths of the present study should be acknowledged. First, the sample size (*n* = 248) was sufficiently large to meet statistical requirements, which enhances the reliability and statistical power of the study results. Second, a comprehensive analytical approach was adopted, including univariate analysis, Pearson correlation analysis, and multiple linear regression analysis. This multi-step analytical strategy enabled systematic exploration of the influencing factors of caregivers’ hope level, providing robust and in-depth evidence for the formulation of targeted intervention measures. Third, the study employed a validated and reliable Hope Level Scale (Cronbach's *α* coefficient: 0.85–0.92 in previous validation; 0.87 in the present study), ensuring the accuracy and validity of the outcome measurement and laying a solid foundation for the credibility of the research findings.

Certain limitations inherent to the present study warrant acknowledgment. First, the cross-sectional study design employed herein only permits a snapshot assessment of caregivers’ hope levels and the identification of correlational relationships among variables, rather than enabling definitive conclusions regarding causal links between influencing factors and hope levels. Second, the study sample was recruited exclusively from a single tertiary grade A children's hospital within a specific geographic region, which may introduce potential selection bias and thereby limit the generalizability of the findings to primary caregivers of children with epilepsy in other settings or localities. Third, the scope of variables examined in this study was relatively narrow: the analysis focused predominantly on sociodemographic characteristics, while neglecting several child-specific factors (e.g., frequency of epileptic seizures, disease severity) and caregiver-centric variables (e.g., the caregivers’ own health status, duration of caregiving tenure) that may exert substantial impacts on hope levels. To address these limitations, future research should adopt longitudinal study designs to track fluctuations in caregivers’ hope levels over a 1–2-year timeframe; such an approach would help clarify the temporal dynamics between influencing factors and hope levels, thereby strengthening causal inferences. Additionally, multi-center sampling strategies incorporating geographically diverse cohorts—encompassing urban, suburban, and rural populations across multiple regions—should be implemented to enhance sample representativeness and improve the external validity of study outcomes.

## Conclusion

In summary, this study has analyzed the hope level of 248 primary caregivers of children with epilepsy and its influencing factors, with key findings as follows: the overall hope level of caregivers was moderate, with the Intimate Relationship dimension being the weakest link; age, marital status, residence, educational level, and monthly household income were significant factors affecting caregivers’ hope level, where older age, married status, urban residence, higher education, and better economic conditions were associated with higher hope level. Clinically, targeted countermeasures should be implemented to address these findings, including strengthening psychological support for vulnerable groups (younger, divorced/widowed, and rural caregivers), improving the accessibility of medical and social support resources, conducting tailored health education and positive attitude training, and focusing on enhancing caregivers’ intimate relationships. To further advance this field, future research should adopt longitudinal designs, multi-center diverse sampling, expand the variable framework to include child- and caregiver-specific factors, incorporate mediating/moderating variables, and integrate multiple validated assessment tools, thereby providing more robust evidence for optimizing caregiver support strategies and promoting the well-being of both children with epilepsy and their caregivers.

## Data Availability

The original contributions presented in the study are included in the article/Supplementary Material, further inquiries can be directed to the corresponding author.
